# S100P promotes trophoblast syncytialization during early placenta development by regulating YAP1

**DOI:** 10.3389/fendo.2022.860261

**Published:** 2022-09-14

**Authors:** Hanjing Zhou, Yibin Pan, Weijie Yang, Chenqiong Zhao, Xiaohe Sun, Binbin Hong, Xiaoying Jin, Tai Zhang, Yinli Zhang, Na Liu, Songying Zhang, Haiyan Zhu

**Affiliations:** ^1^ Assisted Reproduction Unit, Department of Obstetrics and Gynecology, Sir Run Run Shaw Hospital, School of Medicine, Zhejiang University, Hangzhou, China; ^2^ Key Laboratory of Reproductive Dysfunction Management of Zhejiang Province, Hangzhou, China

**Keywords:** trophoblast, syncytialization, S100P, YAP1, rpl

## Abstract

Recurrent pregnancy loss (RPL) is a severe complication of pregnancy that is caused by genetic abnormalities, immune dysfunction, aberrant cell biology, and tissue structure destruction. Among which, placental dysfunction is crucial in the pathogenetic progression of RPL. Although some regulatory factors associated with RPL have been reported, the placental changes correlated with RPL still need to be elucidated. Here, we found that a portion of RPL patients presented with low serum and placental S100P expression. Using a human trophoblast stem cell model, we demonstrated that S100P was exclusively expressed in syncytiotrophoblast (ST)-like syncytia (ST(2D)-TS^CT^) and that loss of S100P expression in ST(2D)-TS^CT^ cells impaired β-hCG secretion, leading to syncytialization failure during early placental development. Moreover, we found that S100P is involved in regulating trophoblast syncytialization by downregulating the protein level of Yes-associated protein 1 (YAP1), which plays a pivotal role in maintaining trophoblast stemness. Together, our findings suggest that S100P plays an essential role in regulating trophoblast syncytialization during early placental development in humans *via* YAP1. Additionally, lower serum S100P levels may predict poor pregnancy outcomes and represent a potentially useful marker for evaluating placental biological function during early pregnancy.

## Introduction

Recurrent pregnancy loss (RPL) is defined as two or more consecutive spontaneous abortions of a clinically established intrauterine pregnancies before 20 weeks with the same spouse (1). Treatment of infertility caused by RPL has been a global challenge for decades, and RPL affects 2-5% of all pregnancies (2, 3). RPL is an intractable complication of pregnancy that is caused by a variety of factors, such as abnormal genetic development, immune dysfunction, cervical insufficiency, maternal endocrine abnormalities, infectious diseases, and male factors (4–7), leading to increased risks of affective disturbance, obstetric complications and longer-term health problems (8). The placenta is a crucial organ involved in the pathogenetic progression of RPL.

The syncytial layer, which is located at the outermost surface of the placenta and has a continuous surface measuring 12 to 14 m^2^ at term, is directly bathed in maternal blood and is involved in nutrient exchange and hormone communication between the mother and fetus (9). The development of the syncytial layer initially includes the formation of multinucleated cells by lateral fusion of cytotrophoblast cells (CTBs). This process is completed *via* the fusion of new CTBs for the establishment of the multinucleated cell layer - the syncytiotrophoblast (STB); this process is called syncytialization (10). Unlike other epithelial differentiation processes, trophoblast syncytialization is both an endocrinological event and a cell fusion event (10, 11), and is regulated by various molecular and signaling pathways at the right temporal and spatial point (12). On the one hand, syncytialization results in the secretion of chorionic gonadotropin (CG) subunit proteins encoded by chorionic gonadotropin alpha (*CGA*) and chorionic gonadotropin beta (*CGB*), which maintain ovarian corpus luteum function (13). In addition, syncytialization results in the expression of 11β-HSD2, which is a microsomal enzyme complex that is responsible for the interconversion of cortisol and cortisone and thus protects the embryo from the potentially detrimental effects of cortisol (14). On the other hand, zonula occludens-1 (ZO-1) is located at intercellular boundaries between CTBs and between CTBs and the STB, forms tight junctions, and its expression is substantially decreased during cell fusion (15). Another transmembrane protein, E-cadherin, which is involved in cell-cell adhesion by promoting the formation of adherent junctions, is decreased during syncytialization due to alterations in both F-actin structure and expression (16, 17). However, the underlying mechanisms that are critical to regulating syncytialization are poorly defined.

In 2018, human trophoblast stem (TS) cells were successfully established from human blastocysts (TS^blast^) and proliferative cytotrophoblast (TS^CT^) cells which have the ability to differentiate into extravillous cytotrophoblast (EVT)- and syncytiotrophoblast (ST)-like cells (18, 19). Human TS cells have been demonstrated to be a valuable *in vitro* model for molecular and functional characterization analysis of human trophoblast cells because their transcriptomes and methylome profiles are similar to those of primary trophoblast cells (18, 20). When induced by exogenous agents, TS^CT^ cells cease proliferation and commence differentiation into STB and EVTs. The gene expression profiles of TS^CT^-derived ST-like cells were similar to those of primary ST cells. Therefore, human TS cells are useful for studying placental development and understanding the pathogenesis of placental disorders (18).

S100P is a member of the S100 family. As a 95-amino acid calcium-binding protein involved in calcium signaling transduction, S100P was first purified and characterized from placentas (21–23). The structure of S100P is altered after binding to calcium, resulting in protein activation and the acquisition of a conformation enabling binding of other proteins (24). S100P regulates many intracellular and extracellular activities, including gene transcription, protein phosphorylation, enzyme activities, cytoskeleton component dynamics, cell proliferation and differentiation (25). S100P contains two EF-hand domains. Each domain consists of two helices, and a calcium-binding loop exists between them. S100P plays a key role in the progression of a variety of cancers (26). In lung cancers, S100P increases cell migration, invasion and metastasis by interacting with integrin α7, which activates focal adhesion kinase (FAK) and AKT (27). S100P is overexpressed in colorectal cancer tissues and participates in regulating cell invasion and metastasis by binding to the SLC2A5 promoter, thereby reducing its methylation and activating its transcription (28). Although most studies have focused on the function of S100P in malignant tumors, a relationship between S100P and calcium signaling in mammalian embryo and placental development has also been reported given that S100P promotes cell proliferation, survival, invasion and vascularization (29–32). Although there is a lack of evidence showing that S100P is directly involved in regulating mouse placenta development, calcium signaling is required for trophoblast proliferation, invasion, differentiation, and fusion during mouse placentation (23). In humans, recent studies have indicated that S100P is highly expressed in the human endometrium during the “implantation window” (33–35). As gestation progresses, S100P is strongly expressed on the syncytial layer of the human placenta, and this expression lasts throughout gestation (36). In addition, the transient receptor potential (TRP) channel protein TRPV6, which plays a vital role in calcium transport, is expressed in human CTBs, and this expression increased during syncytialization (37). Furthermore, S100P enhanced the motility and invasion of human trophoblast cell lines (38) and was able to regulate trophoblast-like cell proliferation *via* the P38 mitogen-activated protein kinase (MAPK) pathway (39).

The Hippo signaling pathway is a highly conserved signaling pathway that participates in a variety of physiological processes, including organ size control, tissue regeneration, cell stemness and differentiation (40–43). The Hippo signaling pathway is activated by numerous triggers, such as high cell density, G protein-coupled receptor signaling, and cell polarity, provoking phosphorylation of macrophage-stimulating protein kinases 1/2 (MST1/2) and large tumor suppressor kinases 1/2 (LATS1/2) (44). Yes-associated protein 1 (YAP1), a key component of the Hippo signaling pathway, interacts with the PDZ-binding motif (TAZ), resulting in its inactivation by either degradation or cytoplasmic retention. Conversely, when the Hippo signaling pathway is “off”, YAP1 is dephosphorylated and translocates to the nucleus, where it maintains stemness and promotes proliferation by acting as a coactivator of TEA domain (TEAD) transcription factor families. The Hippo signaling pathway could be a main driver of placental development (45). Intriguingly, YAP/TEAD4 transcriptional complexes play vital roles in murine trophectoderm (TE) development in preimplantation embryos by activating *Cdx2* (46, 47). Regarding the importance of YAP/TEAD4 complexes for trophoblast development in postimplantation embryos, recent studies have demonstrated that both YAP1 and TEAD4, which are specifically conserved in the CTB progenitors of human first-trimester placentas, are crucial for the self-renewal of TS cells and the proliferation of progenitors (48).

S100P expression is regulated by a variety of transcription factors. It has been reported that the SMAD and STAT families are promoters of S100P that can upregulate S100P expression (49). On the one hand, inhibition of the kinase Warts (Wts), an upstream factor of the Hippo signaling pathway, or activation of the downstream transcriptional cofactor Yorkie (Yki) reduces the expression level of STATs (50). On the other hand, SMADs have also been confirmed to be negatively regulated by YAP/TAZ, transcriptional cofactors downstream of the Hippo pathway (51, 52). Research has shown that the YAP/TEAD complexes suppress lung and breast cancer metastasis by sequestering S100P *via* the lncRNA *NORAD* (31). Furthermore, it has been demonstrated that the induction of S100A7, a member of the S100 family, can be significantly inhibited by nuclear YAP, and TEAD1 is required for YAP transcriptional repression of S100A7 (53, 54).

Although a variety of studies have shown that S100P is regulated by YAP1 of the Hippo signaling pathway in different tissues and organs, the relationship between S100P and YAP1 in the human placenta and their roles in regulating trophoblast syncytialization remain unclear. Therefore, in this study, we aimed to investigate the vital role of S100P in trophoblast syncytialization during early gestation. We used human trophoblast stem cells and gain- and loss-of-function methods to evaluate S100P-mediated molecular mechanisms involved in trophoblast syncytialization. Furthermore, using patient-derived villi and serum, we tested whether downregulation of S100P is associated with idiopathic RPL. Our analyses demonstrated an underlying mechanism in which S100P regulates trophoblast syncytialization during early gestation by inhibiting YAP1 to induce placentation and ensure progression of pregnancy.

## Materials and methods

### Human samples

The first-trimester human placenta tissues of RPL patients and healthy controls (HCs) were collected from women undergoing dilatation and curettage at the sixth to eighth week of gestation in the Department of Obstetrics and Gynecology of the Sir Run Run Shaw Hospital affiliated to the School of Medicine, Zhejiang University. The study was reviewed and approved by the Ethics Committee of Sir Run Run Shaw Hospital affiliated to the School of Medicine, Zhejiang University. All participants were provided with written informed consent to participate in this study.

The HCs were included women who already had a healthy pregnancy at term and opted for an elective abortion at 6-8 weeks in a subsequent apparently healthy pregnancy. Patients suffering from RPL were included if they had an empty gestational sac or embryo stoppage confirmed by type-B ultrasound scans at least twice, containing the current pregnancy. The exclusion criteria for RPL were (i) genital malformation; (ii) abnormal karyotype of parents or abortuses; (iii) endocrine or metabolic disorders; (iv) autoimmune diseases; (v) other major diseases such as hypertension and cancers; and (vi) improper drug treatment, exposure to chemicals or radiation (55). A total of 16 HC and 16 RPL first-trimester placentas were collected. The clinical characteristics of the recruited patients are provided in **Table 1**.

**Table 1:** Clinical characteristics of the recruited patients.

	Healthy controls	Recurrent pregnancy losses	*p* value
Sample size	16	16	
Maternal age (years)	25.8 ± 2.6	28.4 ± 2.3	ns
Gestational age at D&C (days)	48 ± 3.8	50 ± 3.4	ns
Number of spontaneous abortions	n/a	2.5 ± 0.63	n/a

Data are mean values ± SEM. D&C, dilatation and curettage; ns, not significant; n/a, not applicable.

### Isolation, differentiation and identification of human trophoblast cells

The isolation and culture of human TS^CT^ cells were performed following a published protocol (18). Three first-trimester placental villi from healthy mothers were collected. The sex and developmental stage of each human sample used for the derivation of TS^CT^ cells are summarized in **Supplementary Table SI**. In brief, the first-trimester placental villi were collected and cut into small pieces. All tissues were enzymatically digested thrice in a mixture containing equal amounts of TrypLE (Thermo Fisher Scientific, USA) and Accumax (Innovative Cell Tech, USA) for 20 min at 37°C. Cell suspensions were then filtered through a 70 μm mesh filter (352350, BD Falcon, USA). ITGA6 is a widely used cell lineage marker for CTBs (56). CTBs were immunomagnetically purified using an EasySep phycoerythrin (PE)-positive selection kit (Stemcell Technologies, Canada) and a PE-conjugated anti-ITGA6 antibody (130-097-246, Miltenyi, Germany). The selected cells were seeded in a 6-well plate (Corning, USA) coated with 5 μg/mL collagen IV (354233, Corning, USA) at a density of 0.5-1×10^6^ cells per well and cultured in 2 mL of TS medium. CTBs were dissociated with TrypLE for 10-15 min at 37°C. The cells at passages 10-30 were used for analyses and differentiation assays. Information regarding the TS medium is presented in **Supplementary Table SII**.

For ST(2D)-TS^CT^ cell differentiation, TS^CT^ cells were collected and then seeded into a 6-well plate precoated with 2.5 mg/mL collagen IV at a density of 1×10^5^ cells per well. The ST(2D) medium was replaced at day 3, and the culture was continued for 3 days.

To assess ST(3D)-TS^CT^ cell differentiation, TS^CT^ cells were seeded in 6-cm Petri dishes and cultured in 3 mL of ST(3D) medium. An equal amount of fresh ST(3D) medium was added at day 3, and the cells were collected at day 6 after they were passed through a 40-μm mesh filter (352340, BD Falcon, USA) to remove dead cells and debris. The cells remaining on the 40-μm mesh filter were used for further research.

For the induction of EVT-TS^CT^ cells, TS^CT^ cells were seeded in 6-well plates precoated with 1 mg/mL collagen IV at a density of 0.75×10^5^ cells per well. Matrigel (354234, Corning, USA) was added to a final concentration of 2% immediately after suspending TS^CT^ cells in the precooled medium. The medium was replaced with EVT medium without NRG1 on day 3, and Matrigel was added to a final concentration of 0.5%. After the cells reached 80% confluence at day 6, they were dissociated with TrypLE for 15-20 min at 37°C and passaged to a new collagen IV precoated 6-well plate at a 1:2 split ratio. The cells were suspended in the EVT medium without NRG1 and KSR, and Matrigel was added to a final concentration of 0.5%. EVT-TS^CT^ cells were collected and analysed after two additional days. Information regarding ST(2D), ST(3D) and EVT media is presented in **Supplementary Table SII**.

The morphologies of TS^CT^, ST(2D)-TS^CT^ and EVT-TS^CT^ cells were captured by an inverted microscope Primovert with a 10x objective using bright field microscopy (Zeiss, Germany).

### Measurement of β-hCG and S100P levels

The supernatant of TS cells was collected as a control. The ST(2D)-TS^CT^ cell medium was replaced on day 3 and collected on day 3 and 6. All supernatants were centrifuged at 10000 × g for 10 minutes to remove impurities before further research. The levels of secreted β-hCG and secreted S100P were measured using an hCG enzyme-linked immunosorbent assay (ELISA) kit (ABN-KA4005, Abnova, Japan) and a S100P ELISA kit (ABN-KA0093, Abnova, Japan), respectively. Total protein was extracted from cells using Radio Immunoprecipitation Assay (RIPA) buffer (P0013B, Beyotime, China) at 4°C for 30 minutes and measured with a Pierce Bicinchoninic Acid (BCA) Protein Assay Kit (23225, Thermo Scientific, USA). The final concentrations of secreted β-hCG and secreted S100P were normalized to the total protein concentration. Serum secreted S100P levels were measured with a S100P ELISA kit (ABN-KA0093, Abnova, Japan) following the instructions.

### Real-time quantitative PCR analysis

Total RNA samples were isolated from TS^CT^ and ST(2D)-TS^CT^ cells using an RNA-Quick Purification Kit (RN001, ES Science, China) in accordance with the manufacturer’s protocol. Total RNA samples (1 μg) were reverse-transcribed with HiScript II Reverse Transcriptase (R201-1, Vazyme, China). SYBR Green Supermix (D7260, Beyotime, China) and the CFX96 system (Bio-Rad, USA) were used for real-time PCR. The relative gene expression levels in the experimental samples were compared with those in the control samples, and all experiments were performed in triplicate. The primer sequences are listed in **Supplementary Table SIII**.

### Western blotting

Protein samples were extracted from tissues and cells using ice-cold RIPA lysis buffer containing 1% phenylmethanesulfonyl fluoride (PMSF) (ST506, Beyotime, China). Total prepared protein samples were separated by sodium dodecyl sulfate-polyacrylamide gel electrophoresis (SDS-PAGE) (PG113 and PG114, Epizyme Biotech, China) and then transferred to polyvinylidene difluoride (PVDF) membranes (Millipore, USA). After blocking with 5% skim milk (232100, BD Difco, USA) at room temperature for at least 1 hour, the membranes were incubated with primary antibodies overnight at 4°C. The following primary antibodies were used in the experiments: S100P (ab133554, 1:1000, Abcam, USA), TEAD4 (ab58310, 1:1000, Abcam, USA), β-hCG (sc271062, 1:300, Santa Cruz, USA), YAP1 (sc101199, 1:500, Santa Cruz, USA), 11β-HSD2 (sc365529, 1:500, Santa Cruz, USA), E-cadherin (3195, 1:1000, CST, USA), ZO-1 (13663, 1:1000, CST, USA), and GAPDH (60004-1-Ig, 1:1000, Proteintech, China). After washing in Tris-buffered saline with Tween 20 (TBST) buffer, membranes were incubated with horseradish peroxidase-conjugated anti-rabbit (7074, 1:3000, CST, USA) or anti-mouse IgG secondary antibodies (7076, 1:3000, CST, USA), and visualized by enhanced chemiluminescence (WBKLS0500, Millipore, USA). The images were captured using a ChemiDoc Touch Imaging System (Bio-Rad, USA). In addition, other membranes were incubated with preabsorbed goat anti-rabbit IgG H&L IRDye 800 (ab21677, 1:5000, Abcam, USA) or donkey anti-mouse IgG H&L Alexa Fluor 680 (ab175774, 1:5000, Abcam, USA) secondary antibodies and then visualized using an Odyssey Infrared Laser Imaging System (Licor Bioscience, USA). ImageJ software was used to quantify the intensities of the Western blotting bands.

### Immunofluorescence staining and fusion index calculation

Cells were fixed with 4% paraformaldehyde (MA0192, Meilunbio, China) for at least 30 min and then permeabilized with 0.3% Triton X-100 (T8787, Sigma-Aldrich, USA) for 5 min at room temperature. The frozen sections were permeabilized with 0.3% Triton X-100 for 10 min at room temperature. After blocking in 2% goat serum for 1 hour at room temperature, cells or sections were incubated with primary antibodies overnight at 4°C. The following primary antibodies were used: S100P (ab133554, 1:100, Abcam, USA), β-hCG (ab9582, 1:50, Abcam, USA), YAP1 (sc101199, 1:50, Santa Cruz, USA), TEAD4 (ab58310, 1:50, Abcam, USA), HLA-G (ab7758, 1:50, Abcam, USA), E-cadherin (3195, 1:50, CST, USA), and ZO-1 (13663, 1:50, CST, USA). After washing with TBST buffer, cells or sections were incubated with Alexa Fluor 488- or Alexa Fluor 568-conjugated goat secondary antibody (A11001; A10037; A11034; A11036, 1:500, Invitrogen, USA) for 1 hour at room temperature. The nuclei were stained with Hoechst 33342 Stain Solution (H3570, 1:1000, Invitrogen, USA) for an additional 10 min, and images were captured with a laser scanning confocal microscope (Zeiss, LSM800, Germany).

The fusion index is calculated as the ratio of the number of nuclei in the syncytia divided by the total number of nuclei, and the number of nuclei in the syncytia, defined as nuclei at least three are surrounded by a cell membrane (11). Cell boundaries were marked by ZO-1 (13663, 1:50, CST, USA). The total number of nuclei and the number of nuclei in the syncytia were counted in each slide. At least ten images from each group were captured and analyzed. All slides were counted by two independent individuals for comparison.

### Immunohistochemistry staining of paraffin sections

Paraffin slides were dewaxed with xylene for 20 min and 100%, 95%, and 75% ethanol in sequence for a total of 30 min and boiled in Antigen Retrieval Buffer (citrate buffer, pH 6.0, or Tris-EDTA buffer, pH 9.0) (ab93678 and ab93684, Abcam, USA) at 100°C for 15 minutes. The slides were blocked with 2% goat serum for 1 hour at room temperature and incubated with primary antibodies at 4°C overnight. The following primary antibodies were used: S100P (ab133554, 1:100, Abcam, USA) and β-hCG (ab9582, 1:50, Abcam, USA). After washing with TBST buffer, the slides were incubated with horseradish peroxidase (HRP)-conjugated secondary antibodies for 1 hour at room temperature (GK500710, Gene Tech, China). HRP-conjugated antibodies were detected with diaminobenzidine (DAB) (GK500710, Gene Tech, China) for 10 min. The nuclei were then stained with hematoxylin solution (ab220365, Abcam, USA) for an addition 5 min. Images were captured with an Axio Scope A1 (Zeiss, Germany).

### Small interfering RNA transfection

To knockdown *S100P* expression, TS^CT^ and ST(2D)-TS^CT^ cells were cultured in 12-well plates and transfected with small interfering RNAs (siRNAs) targeting *S100P* (siS100P) or negative control siRNA (siNC) with Lipofectamine™ 3000 Transfection Reagent (L3000015, Thermo Fisher Scientific, USA) following the manufacturer’s instructions. The siRNAs were purchased from Ribo Bio Co., Ltd. (Guangzhou, China). The siS100P sequence was 5-CAAGGATGCCGTGGATAAA-3, and the negative control sequence was 5-GATCATACGTGCGATCAGA-3.

### Overexpression in TS^CT^ cells

Cell lines overexpressing *S100P* were generated by infection with a lentiviral vector (CMV-MCS-3FLAG-Ubi-ZSGreen-IRES-Puro) carrying the *S100P* cDNA sequence; the vector was purchased from Guannan Bio Co., Ltd. (Hangzhou, China). TS^CT^ cells were seeded in six-well plates at 80-90% confluence the day before infection. The next day, TS^CT^ cells were infected with lentivirus in culture medium for 24 hours. Then, the medium was replaced before treatment. Approximately 80% of the cells were infected with overexpression lentivirus. Successful infection was confirmed by qPCR and Western blotting assessments of S100P levels. For long-term culture, clones were maintained in the presence of 2 µg/mL puromycin.

The recombinant human S100P protein (12635-HNAE, Sino Biological, China) consisted of 95 amino acids and had a calculated molecular mass of 10.4 kDa. The recombinant protein was first diluted with ultrapure water at room temperature and then added to TS^CT^ cell culture medium at concentrations of 5, 10, 15, and 20 μM for 24 hours.

### RNA-seq

TS^CT^ cells were seeded into 10-cm cell culture dishes and differentiated into ST(2D)-TS^CT^ cells at a 1:1 ratio. On day 1, the ST(2D)-TS^CT^ cells were treated with siRNA targeting *S100P* or negative control as described above. After 4 days, the cells were harvested and total RNA from each group was extracted with TRIzol reagent (Vazyme, China). Three replicate samples were collected for each group. The samples were then processed for reverse transcription, cDNA purification and library construction by Applied Protein Technology company (Shanghai, China). The results were analyzed on the Illumina NovaSeq 6000/MGISEQ-T7 platform. Differentially expressed genes were analyzed with significance criteria of log2 (fold change) ≥1 and multi-test adjusted *p* ≤ 0.05 by Cufflinks (version 2.1.1) and R (version 3.5.1). Gene Ontology (GO) and Kyoto Encyclopedia of Genes and Genomes (KEGG) pathway analyses were performed using clusterProfiler (version 3.18.1). The processed RNA-seq data with fragments per kilobase per million (FPKM) values of all genes are presented in **Supplementary Table SIV**.

### Statistical analysis

All the experiments in this study were performed on three TS^CT^ cell lines at least in triplicate. GraphPad Prism 6.0 software was used for statistical analysis. Statistical comparisons between two groups were carried out using unpaired Student’s t test after confirming the normal distribution of the data by one-sample Kolmogorov-Smirnov test for Gaussian distribution or using Mann-Whitney U test for comparisons of data that did not show Gaussian distribution. The comparison of continuous variables among groups was carried out by one-way analysis of variance (ANOVA) followed by least significant difference (LSD) tests. All the data are shown as the mean values ± standard error of the means (SEMs).

## Results

### S100P expression is decreased in the serum and placentas of RPL patients during early gestation

**Figure 1:**
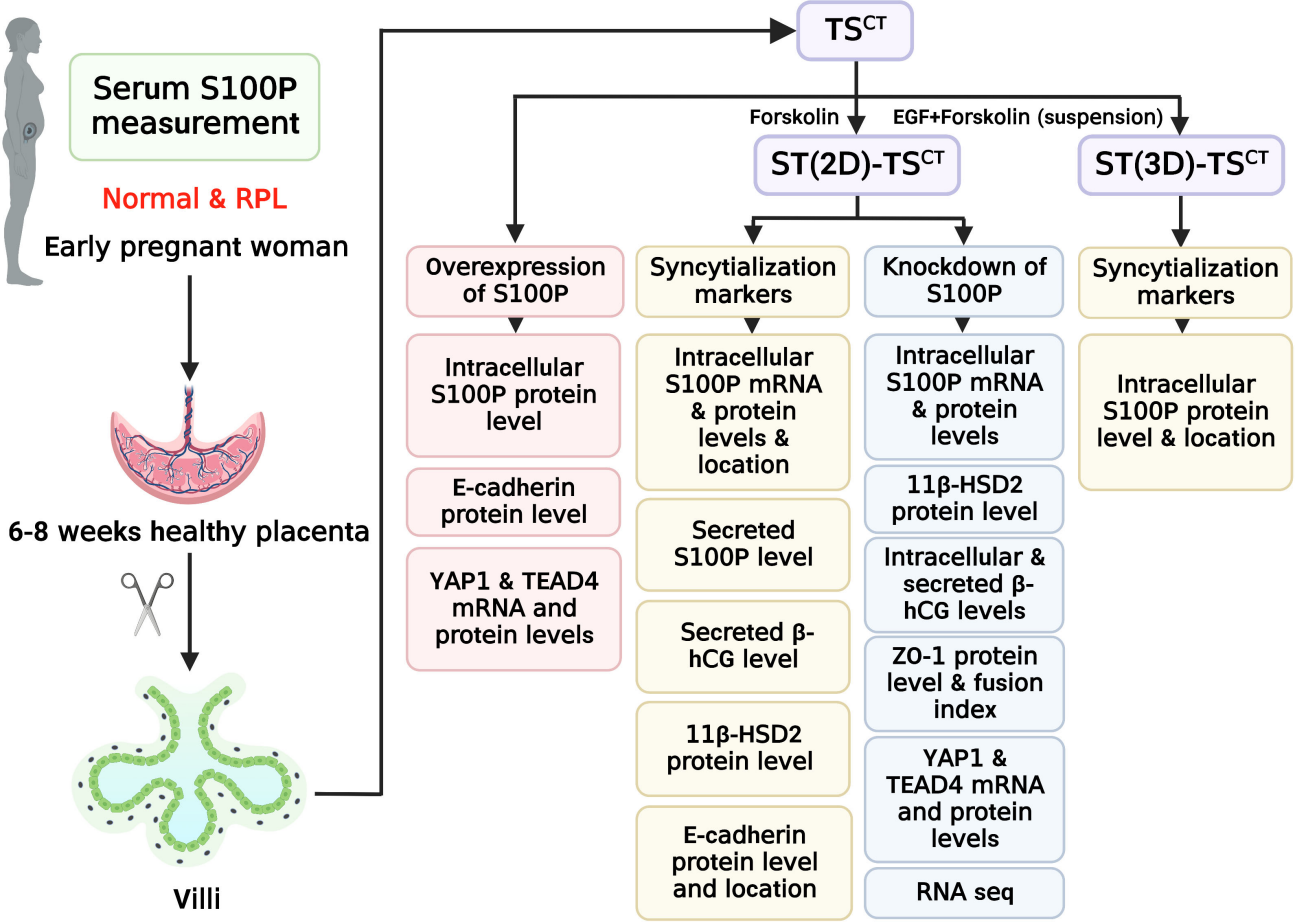
Flowchart of the experimental design. Healthy controls and patients with RPL were chosen based on the inclusion criteria. Villi and serum samples from participants were collected to measure tissue and serum S100P levels, respectively. Human TS^CT^ cells were isolated and cultured from human villous cytotrophoblasts. ST(2D)-TS^CT^ and ST(3D)-TS^CT^ cells were used to investigate intracellular S100P expression and location, whereas ST(2D)-TS^CT^ cells were used to detect 11β-HSD2 protein levels, E-cadherin expression and localization, and secreted S100P and β-hCG levels in the culture medium. After knocking down *S100P* in ST(2D)-TS^CT^ cells, intracellular S100P mRNA and protein levels, 11β-HSD2 protein levels, intracellular and secreted β-hCG levels, ZO-1 expression and localization, the fusion index, YAP1 and TEAD4 mRNA and protein levels, and RNA-seq were detected. After overexpressing *S100P* in TS^CT^ cells, intracellular S100P and E-cadherin protein levels, and YAP1 and TEAD4 mRNA and protein levels were detected.

**Figure 2:**
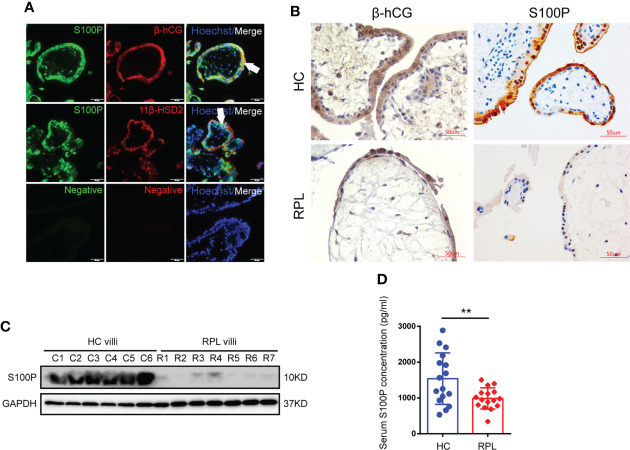
Placenta and serum S100P levels in healthy controls and RPL patients. **(A)** Immunofluorescence staining of S100P (green), 11β-HSD2 (red), β-hCG (red) and Hoechst staining (blue) in human healthy placentas (sixth to eighth week of gestation). White arrows indicate STB. Representative images from three independent experiments are shown. Scale bar, 50 μm. **(B)** Immunohistochemistry staining of S100P in human placentas extracted from women at six- to-eight weeks of gestation. β-hCG was used to identify syncytiotrophoblast layers. Scale bars, 50 μm. **(C)** Representative Western blotting of S100P expression in normal and RPL placental tissues (Ctrl n=6 vs. RPL n=7). **(D)** Measurement of serum S100P levels in healthy controls and RPL patients by ELISA (n=16). **p=0.0079.

We collected villi from HC and RPL women in the sixth to eighth week of gestation and performed the experiments laid out in the flowchart (**Figure 1**). We found that S100P expression was exclusively localized with β-hCG and 11β-HSD2 expression on the syncytial layer of villi from healthy human placentas (**Figure 2A**). To further determine S100P expression levels in placenta tissues, we collected villi from healthy mothers and patients suffering from RPL during early gestation. The immunohistochemistry results showed low S100P and β-hCG expression in the villi of RPL patients (**Figure 2B**), in line with the Western blotting results, which indicated low expression of S100P (**Figure 2C**). As S100P is a secreted calcium-binding protein that can be detected in both human serum and plasma, we measured the serum levels of S100P in patients with RPL and compared them with those in HCs. Interestingly, the serum S100P levels were significantly decreased in patients with RPL (**Figure 2D**).

### S100P is exclusively expressed during trophoblast cell syncytialization *in vitro*

**Figure 3:**
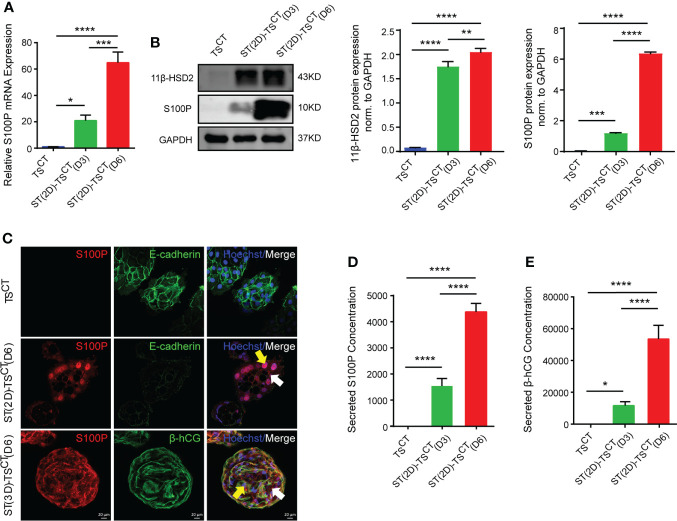
Increased S100P expression during FSK-induced syncytialization of TS^CT^ cells. **(A)** Quantification of *S100P* mRNA expression in ST(2D)-TS^CT^ cells measured on D3 and D6. The mean values ± SEMs (normalized to *GAPDH*) are shown. D3: day 3, D6: day 6. *p=0.0110; ***p=0.0002; ****p<0.0001. **(B)** Western blotting analysis of S100P and 11β-HSD2 in ST(2D)-TS^CT^ cells measured on D3 and D6. Semiquantitative analysis of S100P and 11β-HSD2 protein expression normalized to GAPDH (n=3). **p=0.0073; ***p=0.0006; ****p<0.0001. **(C)** Immunofluorescence staining of S100P (red), E-cadherin (green), β-hCG (green) and Hoechst staining (blue) in human TS^CT^, ST(2D)-TS^CT^ (D6) and ST(3D)-TS^CT^ cells (D6). White arrows indicate cytoplasmic S100P expression, and yellow arrows represent nuclear S100P expression. Representative images from three independent experiments are shown. Scale bar, 20 μm. **(D)** Measurement of the secreted S100P levels in human TS^CT^ and ST(2D)-TS^CT^ cultures (D3 and D6) by ELISA. The levels were measured in three independent experiments. ****p<0.0001. **(E)** Measurement of secreted β-hCG levels in human TS^CT^ and ST(2D)-TS^CT^ cultures (D3 and D6) as assessed by ELISA. The levels were measured in three independent experiments. *p=0.0283; ****p<0.0001.

To detect the role of S100P in syncytialization regulation, we first isolated and identified TS^CT^ cells, which had the ability to differentiate into ST- and EVT- like cells (**Figure S1A**). TEAD4 is a marker for TS^CT^ cells, β-hCG is a marker for ST(2D)-TS^CT^ cells, and human leucocyte antigen-G (HLA-G) is a marker for EVT-TS^CT^ cells (**Figure S1B**). After the successful establishment of the TS^CT^ cell model, we treated human TS^CT^ cells with forskolin (FSK) as previously described (18). Under the presence of FSK for 6 days to fully induce TS^CT^ syncytialization, S100P expression was progressively increased at both the mRNA and protein levels in ST(2D)-TS^CT^ cells, as measured at days 3 and 6 (**Figures 3A**, **B**). The increased expression of 11β-HSD2 indicated successful syncytialization (**Figure 3B**). In addition, S100P was expressed both in the cytoplasm and nucleus of ST(2D)-TS^CT^ and ST(3D)-TS^CT^ cells (**Figure 3C**). Interestingly, the level of secreted S100P was significantly increased during FSK-induced syncytialization *in vitro* (**Figure 3D**), and the level of secreted β-hCG, which is a marker for syncytialization, was also increased (**Figure 3E**). These results indicated that S100P was progressively and exclusively expressed intracellularly and secreted outside the cells during TS^CT^ cell syncytialization. In addition, the localization of S100P in ST(2D)-TS^CT^ cells indicated that it may be involved in different biological processes, such as cellular signaling transduction and gene transcription assistance.

### S100P is involved in regulating trophoblast syncytialization

**Figure 4:**
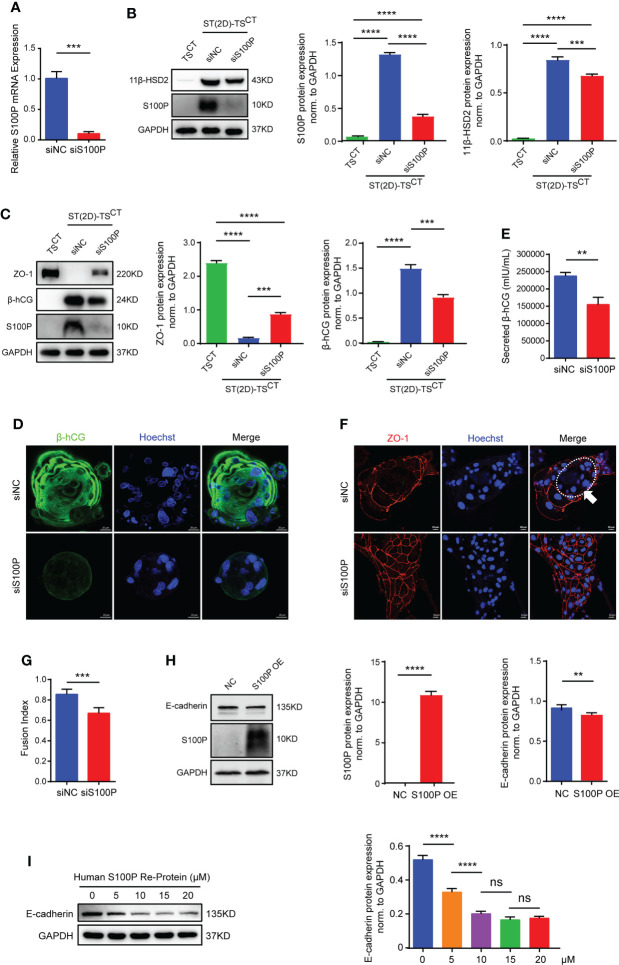
S100P is involved in regulating trophoblast syncytialization. **(A)** Quantification of *S100P* mRNA expression. The mean values ± SEMs (normalized to *GAPDH*) of ST(2D)-TS^CT^ cells (D6) are shown. ***p=0.0002. **(B)** Representative Western blotting of S100P and 11β-HSD2 in siS100P-transfected ST(2D)-TS^CT^ cells (D6). Semiquantitative analysis of S100P and 11β-HSD2 protein expression normalized to GAPDH (n=3). ***p=0.0003; ****p<0.0001. **(C)** Representative Western blotting of ZO-1, β-hCG, and S100P in siS100P-transfected ST(2D)-TS^CT^ cells (D6). Semi-quantification of ZO-1 and β-hCG protein expression normalized to GAPDH (n=3). ***p=0.0001, *p*=0.0003; ****p<0.0001. **(D)** Representative immunofluorescence images stained for the syncytialization marker β-hCG are shown. Hoechst staining was performed to visualize nuclei (n=3, ST(3D)-TS^CT^ cells). Scale bars, 20 μm. **(E)** ELISA measurement of the β-hCG level secreted into the supernatants of the cultures of ST(2D)-TS^CT^ cells (D6) transfected with siS100P. The mean values ± SEMs are shown. **p=0.0038. **(F)** Representative immunofluorescence images of ST(2D)-TS^CT^ cells (D6) transfected with siS100P with ZO-1 (red) and Hoechst staining (blue). The white arrow indicates the fusion area. Representative images of three independent experiments are shown. Scale bar, 20 μm. **(G)** Fusion index of ST(2D)-TS^CT^ cells (D6) transfected with siS100P. The index was assessed in ten images, and the mean values ± SEMs are shown. ***p=0.0002. **(H)** Representative Western blotting of S100P and E-cadherin expression in TS^CT^ cells infected with lentiviral vectors overexpressing *S100P*. Semiquantitative analysis of S100P and E-cadherin protein expression normalized to GAPDH (n=3). **p=0.0084; ****p<0.0001. **(I)** Representative Western blotting of E-cadherin expression in TS^CT^ cells treated with recombinant human S100P protein (5 μM, 10 μM, 15 μM, and 20 μM) (n=3). Semiquantitative analysis of E-cadherin protein expression normalized to GAPDH (n=3). ****p<0.0001; ns: *p*=0.1208, *p*=0.9457.

To determine the biological significance of S100P in human trophoblast cells, we used small interfering RNAs to interfere with *S100P* expression and a lentiviral vector to overexpress *S100P* in human trophoblast stem cells. After transfection followed by differentiation for 6 days, we found a substantial decrease in S100P expression at both the mRNA and protein levels in ST(2D)-TS^CT^ cells (**Figures 4A, B**). Cells transfected with siS100P exhibited a reduction in 11β-HSD2 expression levels by Western blotting (**Figure 4B**), intracellular β-hCG expression by Western blotting and immunofluorescence (**Figures 4C, D**), and secreted β-hCG concentration by ELISA (**Figure 4E**) compared with cells transfected with the control siRNA. ZO-1 expression was significantly increased in ST(2D)-TS^CT^ cells with *S100P* knockdown (**Figure 4C**, representative images are shown in **Figure 4F**), while the fusion index was decreased (**Figure 4G**). Conversely, neither overexpression of the *S100P* gene nor exogenous addition of recombinant human S100P protein triggered syncytialization of TS^CT^ cells, whereas E-cadherin protein expression was decreased (**Figures 4H, I**). These results demonstrated that S100P was involved in regulating the trophoblast syncytialization process, but failed to directly trigger TS^CT^ cell syncytialization even if the expression of the cell-cell adhesion protein E-cadherin was significantly decreased after *S100P* was overexpressed.

### YAP1 maintains the progenitor status of CTB-derived human trophoblast stem cells

**Figure 5:**
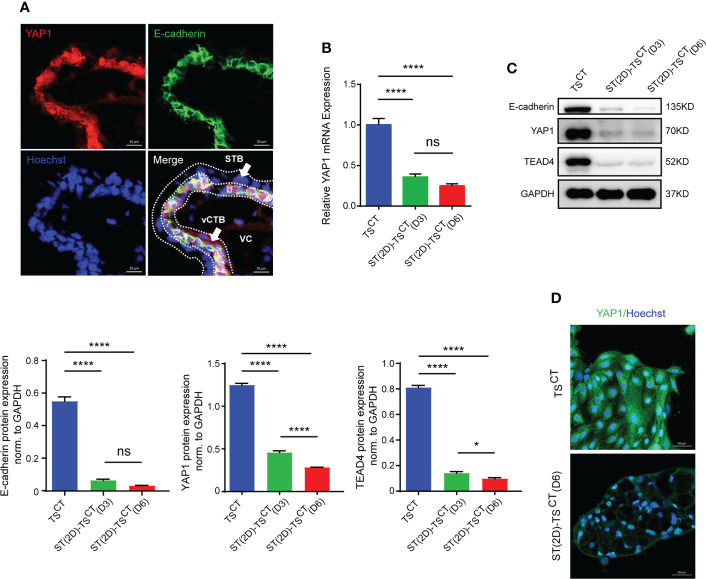
YAP1 and TEAD4 expression is decreased during trophoblast cell syncytialization *in vitro*. **(A)** Immunofluorescence staining of YAP1 (red), E-cadherin (green) and Hoechst staining (blue) in human healthy placentas (sixth to eighth week of gestation). White arrows indicate CTBs and STB, respectively. VC, villous core. Representative images from three independent experiments are shown. Scale bar, 20 μm. **(B)** Quantification of *YAP1* mRNA expression. The mean values ± SEMs (normalized to *GAPDH*) of human TS^CT^, ST(2D)-TS^CT^ (D3) and ST(2D)-TS^CT^ (D6) cell cultures, as measured in duplicate, are shown. ****p<0.0001; ns: *p*=0.1255. **(C)** Representative Western blotting of E-cadherin, YAP1, and TEAD4 expression in TS^CT^ and ST(2D)-TS^CT^ cells (D3 and D6). Semiquantitative analysis of E-cadherin, YAP1, and TEAD4 protein expression normalized to GAPDH (n=3). *p=0.0185; ****p<0.0001; ns: *p*=0.1220. **(D)** Immunofluorescence staining of YAP1 (green) and Hoechst staining (blue) in human TS^CT^ and ST(2D)-TS^CT^ cells (D6). Scale bar, 50 μm.

YAP1 colocalized with E-cadherin in the cytotrophoblast layer of human healthy placentas (six-to-eight weeks of gestation) (**Figure 5A**). YAP1 mRNA and protein expression gradually decreased with syncytialization, as measured on day 3 and 6 (**Figures 5B, C**). The expression of E-cadherin and TEAD4, another key component of the YAP1/TEAD4 transcriptional complex that is crucial for the self-renewal of TS^CT^ cells and proliferation of progenitors, also decreased with syncytialization (**Figure 5C**). In addition, YAP1 expression showed a significant decrease on day 6 after syncytialization (**Figure 5D**). These results demonstrated that YAP1 decreased with trophoblast syncytialization.

### Suppression of YAP1 and TEAD4 by S100P is involved in regulating trophoblast syncytialization

**Figure 6:**
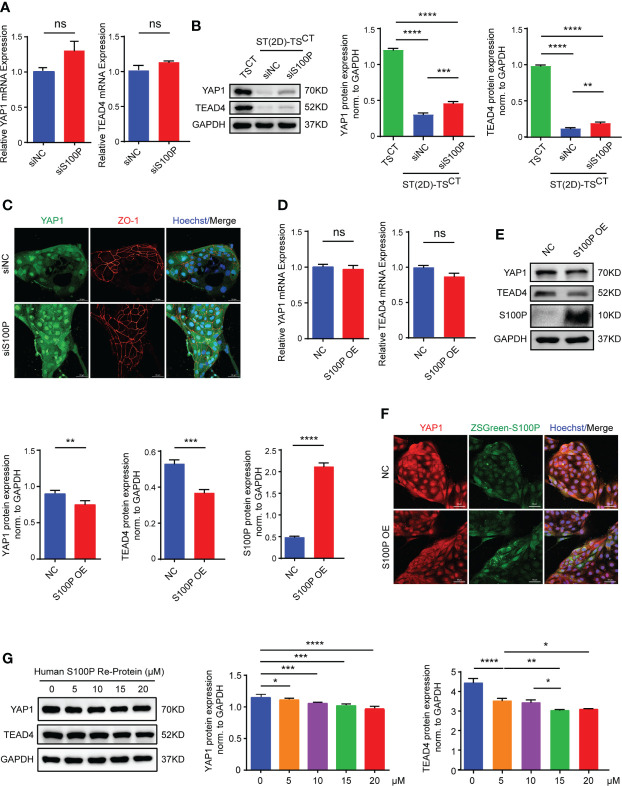
YAP1 and TEAD4 expression are increased in *S100P*-knockdown cells and decreased in S100P-overexpressing cells. **(A)** Quantification of *YAP1* and *TEAD4* mRNA expression. ST(2D)-TS^CT^ cells transfected with siRNA underwent syncytialization for 6 days (n=3). All the bar graphs depict the mean values ± SEMs (normalized to *GAPDH*), and the values were measured in duplicate; ns: *p*=0.0739, *p*=0.1537. **(B)** Representative Western blotting of YAP1 and TEAD4 protein levels in TS^CT^ and ST(2D)-TS^CT^ cells that were transfected with siS100P and underwent syncytialization for 6 days (n=3). Semi quantification of YAP1 and TEAD4 protein expression normalized to GAPDH (n=3). **p=0.0023; ***p=0.0007; ****p<0.0001. **(C)** Representative immunofluorescence images of staining for YAP1 and the TS^CT^ cell boundary marker ZO-1. Hoechst staining indicates nuclei (n=3). Scale bars, 50 μm. **(D)** Quantification of *YAP1* and *TEAD4* mRNA expression was performed by qPCR. TS^CT^ cells were infected with a lentiviral vector inducing overexpression of *S100P* (n=3). All the bar graphs show the mean values ± SEMs (normalized to *GAPDH*). ns: *p*=0.1643, *p*=0.0796. **(E)** Representative Western blotting of YAP1, TEAD4, and S100P expression in *S100P*-overexpressing TS^CT^ cells. Semi quantification of YAP1, TEAD4 and S100P protein expression normalized to GAPDH (n=3). **p=0.0018; ***p=0.0006; ****p<0.0001. **(F)** Representative immunofluorescence images of ZSGreen-S100P and YAP1 staining. Hoechst staining indicates nuclei. Scale bars, 50 μm. **(G)** Representative Western blotting of YAP1 and TEAD4 expression in TS^CT^ cells treated with recombinant human S100P protein (5 μM, 10 μM, 15 μM, and 20 μM) (n=3). Semi quantification of YAP1 and TEAD4 protein expression normalized to GAPDH (n=3). *p=0.0151, *p*=0.0126, *p*=0.0203; **p=0.0051; ***p=0.0005, *p*=0.0001; ****p<0.0001.

We reasoned that *S100P* affects syncytialization by repressing CTB progenitor gene expression or inducing the expression of genes that promote STB formation. First, we detected the mRNA expression levels of a few syncytialization-related genes; interestingly, none of these genes were altered after *S100P* knockdown or overexpression (**Figures S2A, 2B**). Therefore, to identify the potential repressed CTB progenitor gene that mediated *S100P* syncytialization, we examined *YAP1* and *TEAD4* mRNA and protein expression levels after transfection with siRNA targeting *S100P* during TS^CT^ cell syncytialization. Through this analysis, we found that the mRNA levels of *YAP*1 and *TEAD4* were not significantly upregulated (**Figure 6A**), whereas their protein levels were both increased (**Figure 6B**). The immunofluorescence results further confirmed the upregulated expression of *YAP1* and ZO-1 in ST(2D)-TS^CT^ cells transfected with siS100P (**Figure 6C**). Consistently, *YAP1* and *TEAD4* mRNA levels showed no significant change, whereas their protein levels were obviously decreased in *S100P*overexpressing human TS^CT^ cells (**Figures 6D, E**). Immunofluorescence staining also confirmed the decreased YAP1 protein levels (**Figure 6F**). Moreover, TS^CT^ cells treated with the recombinant human S100P protein showed similar results regarding YAP1 and TEAD4 protein levels compared to those with *S100P* gene overexpression (**Figure 6G**). Together, these data suggested that S100P may be involved in regulating trophoblast syncytialization *via* YAP1, which is vital for maintaining the self-renewal capacity of TS^CT^ cells.

### Gene expression profiling of ST(2D)-TS^CT^ cells with *S100P* knockdown

**Figure 7:**
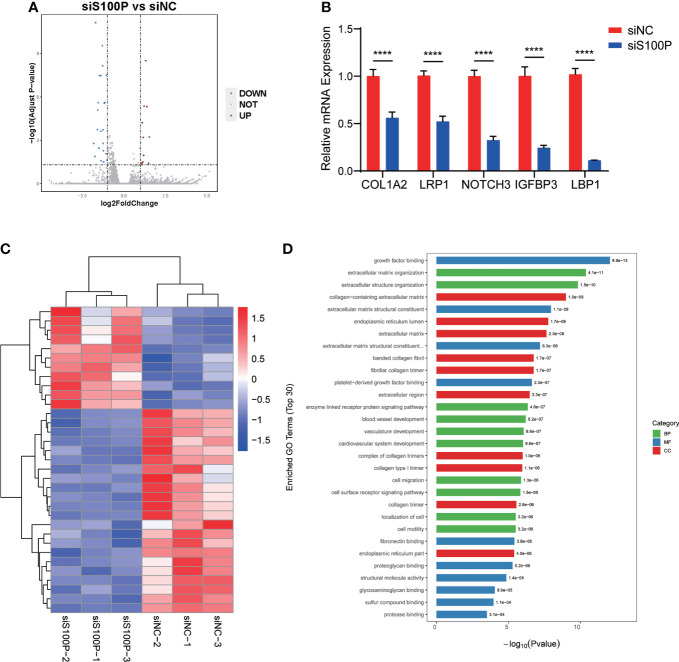
Transcriptional profiling of ST(2D)-TS^CT^ cells with *S100P* interference. **(A)** Volcano plots of differentially expressed genes after *S100P* knockdown. The red dots indicate upregulated genes, and the blue dots indicate downregulated genes. **(B)** RT-qPCR analysis of some downregulated and upregulated genes from the RNA-seq data analysis. Data are presented as the mean values ± SEMs. ****p<0.0001. **(C)** Heatmap of all the upregulated- and downregulated genes between the two groups. **(D)** Enriched biological process, molecular function, and cellular component terms of the downregulated differentially expressed genes in siS100P ST(2D)-TS^CT^ cells as assessed by GO analysis.

To further explore the potential mechanism of S100P regulated trophoblast syncytialization, we performed transcriptome sequencing (RNA-seq) analysis to detect the differentially expressed genes in ST(2D)-TS^CT^ cells with *S100P* knockdown. Data analysis identified 33 significantly differentially expressed genes, 11 of which were upregulated and 22 of which were downregulated in the *S100P* knockdown group compared to the negative control (*p*<0.05 and log2-fold change>1) (**Figure 7A**). We validated the expression of some genes that are known to affect syncytialization, migration and invasion of trophoblast cells by RT-qPCR. We found that *COL1A2, LRP1, NOTCH3, IGFBP3* and *LBP1* gene expression was downregulated, which was consistent with the RNA-seq results (**Figure 7B**). Moreover, the enriched differentially upregulated- and downregulated genes between the two groups of ST(2D)-TS^CT^ cells are shown in the heatmap (**Figure 7C**). We performed GO analysis of the downregulated genes in the *S100P* siRNA interference group, and the results showed specific enrichment of growth factor binding and extracellular matrix organization (**Figure 7D**). Overall, these results demonstrated that the syncytialization of ST(2D)-TS^CT^ cells was negatively regulated after *S100P* knockdown, and extracellular matrix components may play a vital role in the syncytialization process.

## Discussion

S100P is involved in a variety of signal transduction pathways and exerts its biological effects by interacting with its target proteins; however, the mechanism has not been fully elucidated. To date, a number of target proteins that interact with S100P have been found, such as the Receptor of Advanced Glycation End products (RAGE), Calcyclin-Binding Protein (CacyBP/SIP), Ezrin, and Cathepsin D (57). Recent studies have suggested that intracellular S100P exerts its biological effects through Ezrin, whereas the secreted S100P protein mainly acts by binding to RAGE (58). As a temporally and spatially expressed protein, S100P has been demonstrated to play an important role in human embryo and placenta development (59). After the blastocyst is implanted, S100P is first expressed in the TE of the embryo (59). As gestation progresses, S100P becomes highly expressed in placental tissue, and the levels remain elevated throughout the pregnancy cycle (11). S100P affects trophoblast proliferation (60) and enhances the motility and invasion of different trophoblast cell lines (38). In addition, our research found that S100P is also involved in regulating trophoblast syncytialization as knockdown of *S100P* decreased β-hCG secretion and cell boundary stability during the syncytialization process.

S100P expression gradually increased with syncytialization, and reached the maximum level on the sixth day of syncytialization, which was also accompanied by an increase in β-hCG. After knocking down *S100P* for 72 hours from the third day of syncytialization, the levels of intracellular and secreted β-hCG protein and the cell fusion index were significantly decreased on day 6. These results indicated that the deletion of *S100P* affected the trophoblast syncytialization process. Interestingly, the overexpression of the S100P gene and protein in TS^CT^ cells failed to trigger syncytialization; however, the expression of the cell-cell adhesion protein E-cadherin was significantly decreased, which was the same as the decrease in E-cadherin protein expression observed during cell fusion under physiological conditions (61). This result may be attributed to the activation of Ezrin by the S100P protein to reduce the accumulation of E-cadherin in the cell membrane (58, 62).

YAP1 and TEAD4 are key genes involved in maintaining trophoblast cell stemness (45, 48). After knocking down or overexpressing *S100P*, we surprisingly found that YAP1 and TEAD4 were inversely upregulated- or downregulated at the protein level with S100P, whereas no significant alteration in their mRNA levels was observed. In addition, YAP1 and TEAD4 were naturally downregulated with trophoblast syncytialization compared to TS^CT^ cells. *S100P* knockdown partially reversed YAP1 and TEAD4 expression at the protein level in ST(2D)-TS^CT^ cells, but the results did not reach to their levels observed in TS^CT^ cells. This finding indicates that the direct regulation of YAP1 by S100P was not very strong, and S100P may affect YAP1 expression through other pathways. As YAP/TEAD4 complexes repress the expression of STB-associated genes, such as *CGB*, on its promoter regions, it can thereby impair autocrine, hCG-dependent cell fusion and differentiation (45). Thus, we supposed that S100P may indirectly regulate β-hCG expression mediated by YAP1 in a protein-protein interaction manner. Similar results suggesting that S100P participates in cell activities by interacting with its target proteins have been reported in recent years. In hepatocellular carcinoma (HCC), aberrant S100P expression suppresses cell growth and apoptosis by downregulating cyclin D1 and CDK expression at the protein level (63). Moreover, S100P promotes colorectal cancer cell epithelial-to-mesenchymal transition (EMT) as well as migration and invasion by upregulating S100A4 protein levels together with Trx-1 (64). However, it remains unclear how S100P interacts with YAP1 at the protein-interaction level.

Previous studies reported that the YAP/TEAD complexes either sequestered or inhibited S100 family expression, which was contradictory to our findings. In this study, however, the effect of S100P on YAP1/TEAD4 complexes was evident. As all gene expression exhibited strict temporal specificity; *S100P* and *YAP1* were highly expressed at different times during the TS^CT^ cell syncytialization process. Before TS^CT^ cell syncytialization, YAP1 was highly expressed, *S100P* expression was not altered significantly with *YAP1* knockdown because *YAP1* knockdown did not trigger syncytialization induced by FSK. However, after knocking down the highly- expressed *S100P* gene during syncytialization, YAP1 expression was significantly upregulated at the protein level. In carcinoma cell lines, S100P and YAP1 expression levels were both increased under the same treatment, whereas S100P was apparently up- or downregulated after *YAP1* knockdown or overexpression (53, 54). The above findings may differ from the interpretation of the effect of S100P on YAP/TEAD complexes during the trophoblast syncytialization process.

We performed an RNA-seq assay and found that some genes related to trophoblast syncytialization were downregulated after *S100P* interference. It has been demonstrated that trophoblast cell invasion can be enhanced by IGFBP3 upregulation (65). COL1A2 is a crucial component of the extracellular matrix that plays an important role in trophoblast fusion, invasion, angiogenesis and placental barrier maintenance (66–68).

S100P is a secreted calcium-binding protein and has been detected both in human peripheral serum and placentas *in situ* (36). Serum S100P levels are elevated in patients with diabetic peripheral neuropathy and are considered a significant indicator of peripheral neuropathy in patients with type 2 diabetes (69). Moreover, S100P may be recognized as a novel differential diagnostic marker for HCC and a potential predictor of microvascular invasion (MVI) status in HCC patients before surgery. Downregulating S100P in endometrial tissue induces epithelial cell apoptosis, which suggests that S100P is a potential clinical target to improve the success of *in vitro* fertilization (IVF) (70). Our research found reduced S100P expression levels in placentas and serum in RPLs, suggesting that S100P may represent a potential marker for poor pregnancy outcomes.

**Figure 8:**
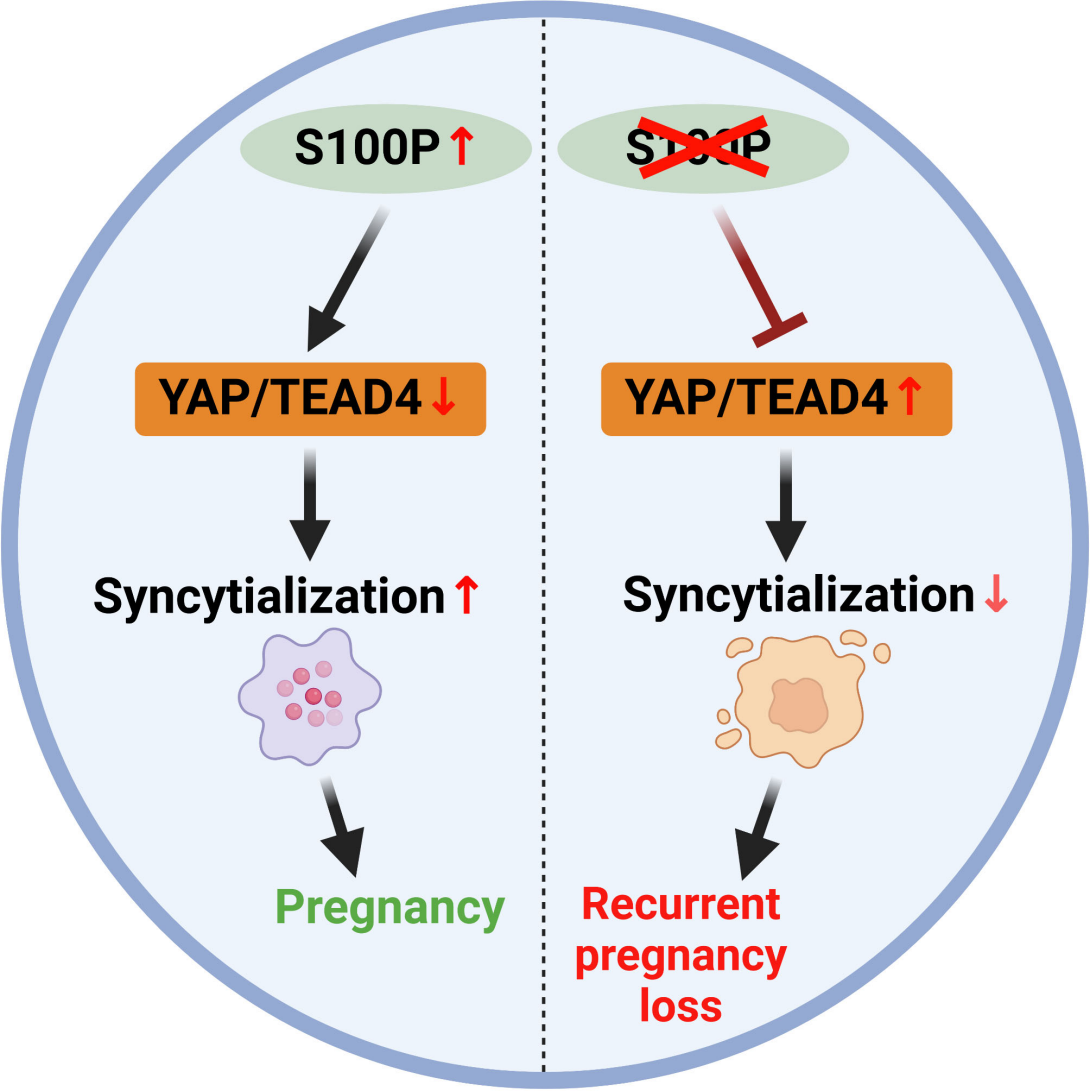
Schematic model illustrating that S100P participates in trophoblast syncytialization during early placenta development by downregulating YAP1 and TEAD4.

In summary, S100P is involved in regulating trophoblast syncytialization by regulating YAP1 at the protein level. Moreover, serum S100P levels are decreased in patients with RPL compared with controls, suggesting that low serum S100P levels are related to poor pregnancy outcomes and may represent a useful marker for evaluating placental biological function during early pregnancy (**Figure 8**). This study demonstrates that S100P plays a pivotal role in trophoblast syncytialization and gestation maintenance by regulating YAP1 and provides a possible approach for predicting early pregnancy maintenance.

## Data availability statement

The datasets presented in this study can be found in online repositories. The names of the repository/repositories and accession number(s) can be found below: NCBI BioProject, PRJNA798551.

## Ethics statement

The studies involving human participants were reviewed and approved by Ethics Committee of Sir Run Run Shaw Hospital, Zhejiang University School of Medicine. The patients/participants provided their written informed consent to participate in this study. Written informed consent was obtained from the individual(s) for the publication of any potentially identifiable images or data included in this article.

## Author contributions

HJZ and YP performed experiments. HJZ, CZ, BH, XJ, TZ and NL collected the clinical samples. HJZ, WY, XS and HYZ analyzed the data. HJZ made the figures and drafted the article. SZ and HYZ critically reviewed the article. SZ and HYZ conceived and supervised the project. All authors contributed to the article and approved the submitted version.

## Funding

This article is supported by the National Key Research and Development Program of China (2018YFC1004800), the National Natural Science Foundation of China (81601236 and 81601308), the Natural Science Foundation of Zhejiang Province (LY19H040009 and LY22H040007).

## Acknowledgments

We are grateful to all patients participated in this research, and we sincerely thank to the help from Department of Obstetrics and Gynecology of the Sir Run Run Shaw Hospital. We thank American Journal Experts from Durham, North Carolina, USA (www.aje.com), for editing the English text of a draft of this manuscript.

## Conflict of interest

The authors declare that the research was conducted in the absence of any commercial or financial relationships that could be construed as a potential conflict of interest.

## Publisher’s note

All claims expressed in this article are solely those of the authors and do not necessarily represent those of their affiliated organizations, or those of the publisher, the editors and the reviewers. Any product that may be evaluated in this article, or claim that may be made by its manufacturer, is not guaranteed or endorsed by the publisher.

## Supplementary material

Supplementary Figure 1 Establishment of human proliferative CT cells in culture**. (A)** Phase contrast images of TS^CT^ cells at postnatal day 6 (P6), ST(2D)-TS^CT^ cells (measured on day 6), and EVT-TS^CT^ cells (measured on day 8). Similar results were obtained with three independent cell lines. D6: day 6, D8: day 8. Scale bars, 50 μm. **(B)** Representative immunostaining images of TEAD4 (CT marker), CGB (ST marker), and HLA-G (EVT marker) in TS^CT^, ST(2D)-TS^CT^ (D6), and EVT-TS^CT^ (D8) cells. The nuclei were stained with Hoechst 33342. Scale bars, 50 μm. Similar results were obtained with three independent cell lines.

Supplementary Figure 2 Alteration of fusion-associated gene expression with S100P knockdown and overexpression. **(A)** qPCR quantification (duplicates) of *CGA, CGB, HSD11β2, GCM-1, ERVW-1*, *ERVFRD-1, ERVV-1* and *ERVV-2* mRNA expression in siS100P-transfected ST(2D)-TS^CT^ cells that underwent syncytialization (6 days) (n=3). All the bar graphs present the mean values ± SEMs (ns, not significant). **(B)** qPCR quantification (duplicates) of *CGA, CGB, HSD11β2, GCM-1, ERVW-1*, *ERVFRD-1, ERVV-1* and *ERVV-2* mRNA expression in TS^CT^ cells infected with lentiviral vectors inducing S100P overexpression. All the bar graphs show the mean values ± SEMs (ns, not significant).
