# Supplementary material for: S100P promotes trophoblast syncytialization during early placenta development by regulating YAP1
Source: Front Endocrinol (Lausanne). 2022 Sep 14;13:860261. doi: 10.3389/fendo.2022.860261 (PMC9515983; doi:10.3389/fendo.2022.860261)
Supplement: Supplementary file 1 [file Table_1.docx]

**Supplementary Table SI** Summary of TS^CT^ Cell Derivation

| TS line | Maternal age (years) | Gestational age at D&C | Karyotype | Doubling time  (Passage number) |
| --- | --- | --- | --- | --- |
| TS^CT^ #1 | 31 | 8 weeks | 46, XY | 25±2.2 hours (P13) |
| TS^CT^ #2 | 24 | 6 weeks | 46, XX | 24±4.7 hours (P13) |
| TS^CT^ #3 | 32 | 7 weeks | 46, XX | 27±1.8 hours (P13) |
