# Supplementary material for: S100P promotes trophoblast syncytialization during early placenta development by regulating YAP1
Source: Front Endocrinol (Lausanne). 2022 Sep 14;13:860261. doi: 10.3389/fendo.2022.860261 (PMC9515983; doi:10.3389/fendo.2022.860261)
Supplement: Supplementary file 2 [file Table_2.docx]

# **Supplementary Table SII** Formula of trophoblast stem cell (TS) medium, ST(2D) medium, ST(3D) medium, and EVT medium supplements added to DMEM/F12 (Thermo Fisher Scientific, USA)

|  | **Component** | **Concentration** | **Identifier and Company** |
| --- | --- | --- | --- |
| TS medium | 2-Mercaptoethanol | 0.1 mM | Cat#21985023  Thermo Fisher Scientific, USA |
|  | FBS | 0.20% (v/v) | Cat#16141-079  Thermo Fisher Scientific, USA |
|  | Penicillin-Streptomycin | 1% (v/v) | Cat#15140122  Thermo Fisher Scientific, USA |
|  | BSA | 0.30% (w/v) | Cat#017-22231  Wako, Japan |
|  | ITS-X supplement | 1% (v/v) | Cat#094-06761  Wako, Japan |
|  | L-ascorbic acid | 1.5 μg/ml | Cat#013-12061  Wako, Japan |
|  | EGF | 50 ng/ml | Cat#053-07871  Wako, Japan |
|  | CHIR99021 | 2 μM | Cat#038-23101  Wako, Japan |
|  | A83-01 | 0.5 μM | Cat#035-24113  Wako, Japan |
|  | SB431542 | 1 μM | Cat#031-24291  Wako, Japan |
|  | VPA | 0.8 mM | Cat#227-01071  Wako, Japan |
|  | Y27632 | 5 μM | Cat#257-00511  Wako, Japan |
| ST(2D) medium | 2-Mercaptoethanol | 0.1 mM | Cat#21985023  Thermo Fisher Scientific, USA |
|  | Penicillin-Streptomycin | 0.5% (v/v) | Cat#15140122  Thermo Fisher Scientific, USA |
|  | BSA | 0.3% (w/v) | Cat#017-22231  Wako, Japan |
|  | ITS-X supplement | 1% (v/v) | Cat#094-06761  Wako, Japan |
|  | Y27632 | 2.5 μM | Cat#257-00511  Wako, Japan |
|  | Forskolin | 2 μM | Cat#067-02191  Wako, Japan |
|  | KnockOut Serum Replacement | 4% (v/v) | Cat#10828028  Thermo Fisher Scientific, USA |
| ST(3D) medium | 2-Mercaptoethanol | 0.1 mM | Cat#21985023  Thermo Fisher Scientific, USA |
|  | Penicillin-Streptomycin | 0.5% (v/v) | Cat#15140122  Thermo Fisher Scientific, USA |
|  | BSA | 0.3% (w/v) | Cat#017-22231  Wako, Japan |
|  | ITS-X supplement | 1% (v/v) | Cat#094-06761  Wako, Japan |
|  | Y27632 | 2.5 μM | Cat#257-00511  Wako, Japan |
|  | Forskolin | 2 μM | Cat#067-02191  Wako, Japan |
|  | KnockOut Serum Replacement | 4% (v/v) | Cat#10828028  Thermo Fisher Scientific, USA |
|  | EGF | 50 ng/ml | Cat#053-07871  Wako, Japan |
| EVT medium | 2-Mercaptoethanol | 0.1 mM | Cat#21985023  Thermo Fisher Scientific, USA |
|  | Penicillin-Streptomycin | 0.5% (v/v) | Cat#15140122  Thermo Fisher Scientific, USA |
|  | BSA | 0.3% (w/v) | Cat#017-22231  Wako, Japan |
|  | ITS-X supplement | 1% (v/v) | Cat#094-06761  Wako, Japan |
|  | Y27632 | 2.5 μM | Cat#257-00511  Wako, Japan |
|  | KnockOut Serum Replacement | 4% (v/v) | Cat#10828028  Thermo Fisher Scientific, USA |
|  | NRG1 | 100 ng/ml | Cat#5218SC  Cell Signaling Technology, USA |
|  | A83-01 | 7.5 μM | Cat#035-24113  Wako, Japan |
