# Supplementary material for: S100P promotes trophoblast syncytialization during early placenta development by regulating YAP1
Source: Front Endocrinol (Lausanne). 2022 Sep 14;13:860261. doi: 10.3389/fendo.2022.860261 (PMC9515983; doi:10.3389/fendo.2022.860261)
Supplement: Supplementary file 3 [file Table_3.docx]

# **Supplementary Table SIII** Primer Sequences for quantitative real time polymerase chain reaction (qRT-PCR)

| Description | Forward | Reverse |
| --- | --- | --- |
| S100P | ATGACGGAACTAGAGACAGCCATGGGC | GGAATCTGTGACATCTCCAGGGCATCA |
| YAP1 | TAGCCCTGCGTAGCCAGTTA | TCATGCTTAGTCCACTGTCTGT |
| COL1A2 | CAGCAGGAGGTTTCGGCTAA | TGCAAGCAGCAACAAAGTCC |
| LRP1 | AGCCAGCTATGCACCAACAC | CCTTGCAGGAGCGGTTATC |
| NOTCH3 | TGGCGACCTCACTTACGACT | CACTGGCAGTTATAGGTGTTGAC |
| IGFBP3 | AGACACACTGAATCACCTGAAGT | AGGGCGACACTGCTTTTTCTT |
| LBP1 | GAAGTCAAGGTCGGTTAGACCC | CATTCTCGCTTGCACTGCTT |
| CGA | TTTCTGGTCACATTGTCGGT | TGGGCAATCCTGCACATCAG |
| CGB | CGGGACATGGGCATCCAA | GCGCACATCGCGGTAGTT |
| ERVW-1 | GGAGGAGATGTGGCACCATT | CCTTCCCACCACAGAAGACC |
| ERVFRD-1 | CCAAATTCCCTCCTCTCCTC | CGGGTGTTAGTTTGCTTGGT |
| ERVV-1 | TAACAGTGGGGCGATAGAGG | AGACTTCACAGCCTCCCAAA |
| ERVV-2 | CAGGCACAGTGGAATGAAAA | GACCTGGTGATGAAGTTGTGG |
| 11β-HSD2 | GACATGCCATATCCGTGCTT | GCTGGATGATGCTGACCTTG |
| GCM1 | TTCCCGGTCACCAACTTCTG | GTAAACTCCCCTGACTTTGTGTT |
| GAPDH | CAGGAGGCATTGCTGATGAT | GAAGGCTGGGGCTCATTT |
