# Supplementary figures and images for: S100P promotes trophoblast syncytialization during early placenta development by regulating YAP1
Source: Front Endocrinol (Lausanne). 2022 Sep 14;13:860261. doi: 10.3389/fendo.2022.860261 (PMC9515983; doi:10.3389/fendo.2022.860261)

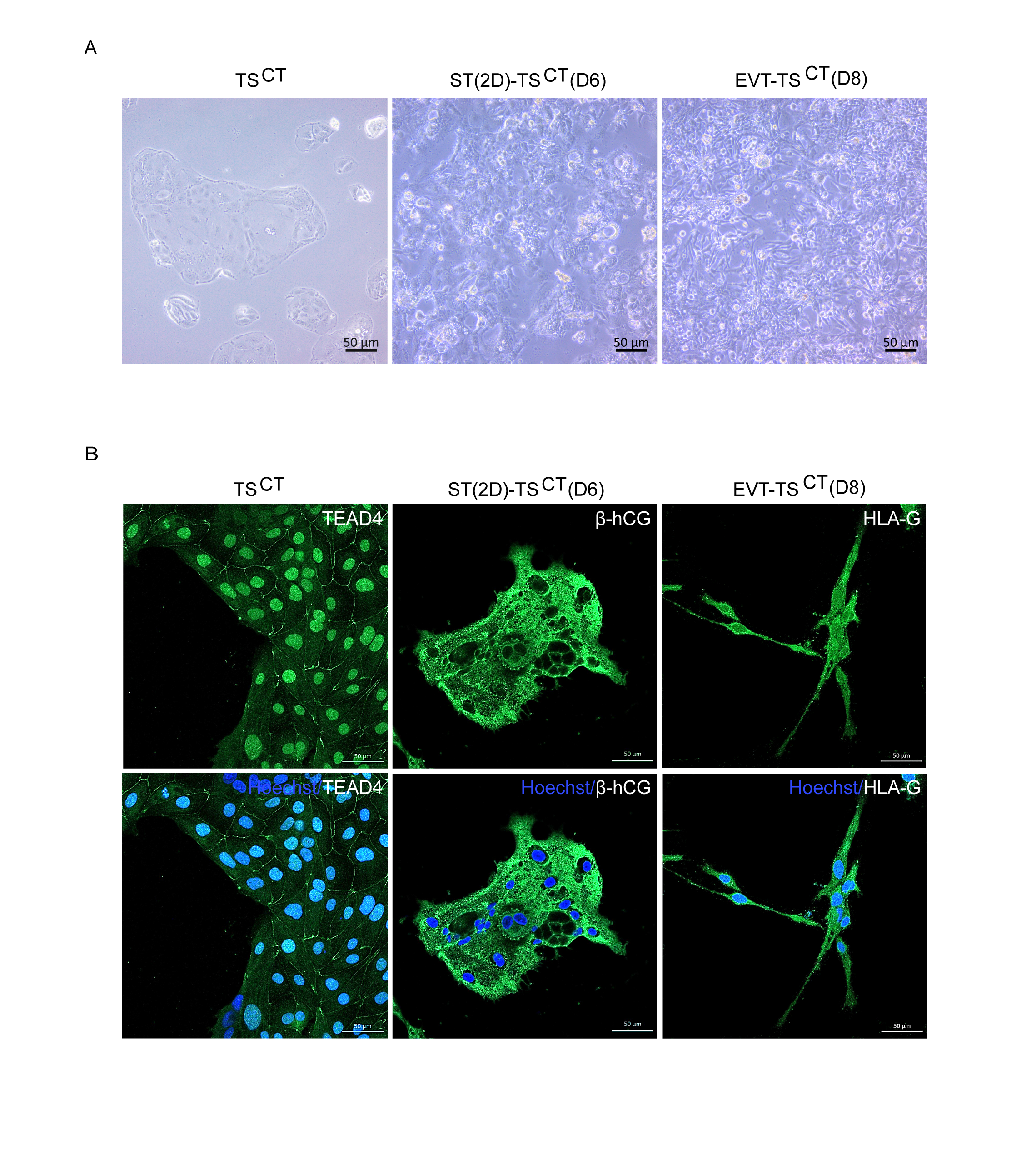

Supplement: Supplementary Figure 1 — Establishment of human proliferative CT cells in culture. (A) Phase contrast images of TSCT cells at postnatal day 6 (P6), ST(2D)-TSCT cells (measured on day 6), and EVT-TSCT cells (measured on day 8). Similar results were obtained with three independent cell lines. D6: day 6, D8: day 8. Scale bars, 50 μm. (B) Representative immunostaining images of TEAD4 (CT marker), CGB (ST marker), and HLA-G (EVT marker) in TSCT, ST(2D)-TSCT (D6), and EVT-TSCT (D8) cells. The nuclei were stained with Hoechst 33342. Scale bars, 50 μm. Similar results were obtained with three independent cell lines. [file Image_1.tif]

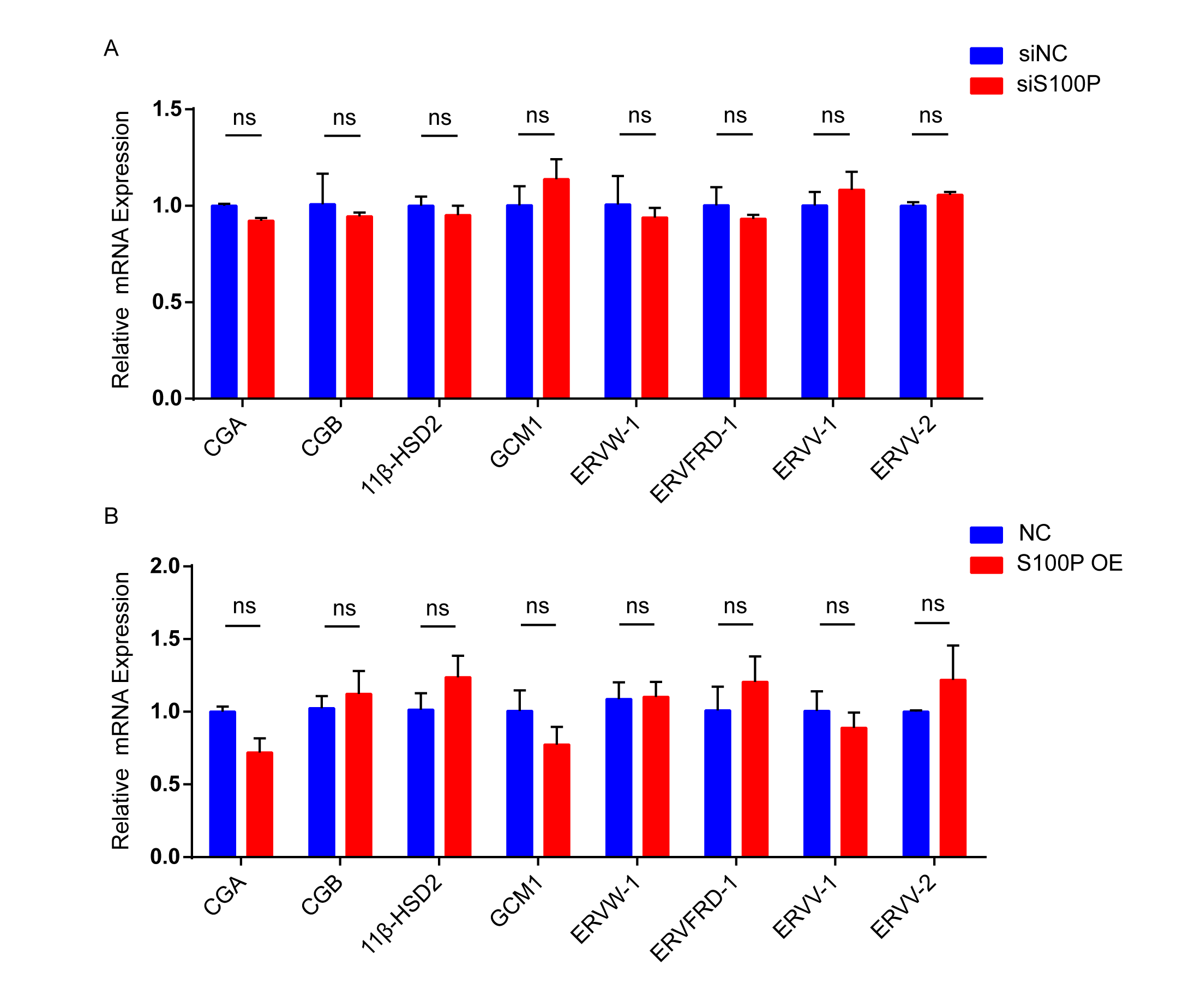

Supplement: Supplementary Figure 2 — Alteration of fusion-associated gene expression with S100P knockdown and overexpression. (A) qPCR quantification (duplicates) of CGA, CGB, HSD11β2, GCM-1, ERVW-1, ERVFRD-1, ERVV-1 and ERVV-2 mRNA expression in siS100P-transfected ST(2D)-TSCT cells that underwent syncytialization (6 days) (n=3). All the bar graphs present the mean values ± SEMs (ns, not significant). (B) qPCR quantification (duplicates) of CGA, CGB, HSD11β2, GCM-1, ERVW-1, ERVFRD-1, ERVV-1 and ERVV-2 mRNA expression in TSCT cells infected with lentiviral vectors inducing S100P overexpression. All the bar graphs show the mean values ± SEMs (ns, not significant). [file Image_2.tif]
